# Modeling species co‐occurrence effects to inform invasive barred owl management and recovery of the northern spotted owl

**DOI:** 10.1002/eap.70195

**Published:** 2026-03-12

**Authors:** Vaibhava Srivastava, Nicholas J. Van Lanen, Rana D. Parshad

**Affiliations:** ^1^ Department of Mathematics Iowa State University Ames Iowa USA; ^2^ US Geological Survey, Fort Collins Science Center Fort Collins Colorado USA; ^3^ Present address: Department of Mathematics and Statistics University of Massachusetts Amherst Amherst Massachusetts USA

**Keywords:** barred owl (*Strix varia*), co‐occurrence effect, fear effect, Lotka–Volterra competition model, population dynamics, spotted owl (*Strix occidentalis*)

## Abstract

Robust estimation of wildlife populations represents a cornerstone of wildlife research and provides critical information to guide management, including identifying at‐risk species, setting harvest rates, and evaluating predator and invasive species control programs. Efforts to enhance population estimation have long included influences one species may have on another, beginning with direct effects of predation on prey populations. More recently, researchers have incorporated co‐occurrence effects, such as fear of a competitor, into Lotka–Volterra competition models to generate more robust wildlife population estimates. Here, we introduce two modified Lotka–Volterra competition models, which incorporate one‐ and two‐way co‐occurrence effects, to estimate populations of two competing species. Using the test case of northern spotted (*Strix occidentalis caurina*) and barred owl (*Strix varia*) populations in the Pacific Northwest region of the United States, we evaluate if these new co‐occurrence models can generate more robust population estimates than previous models. We then evaluate if potential co‐occurrence effects among barred and northern spotted owls are uni‐ or bidirectional. Lastly, we leverage the best‐performing model to evaluate the degree to which a recently proposed barred owl culling program may help recover northern spotted owl populations. Our model results suggest that incorporating co‐occurrence effects improves model fit compared to classical Lotka–Volterra competition models. We found strong evidence for unidirectional co‐occurrence effects of barred owls on northern spotted owls, but not vice versa. Our simulations of barred owl culling suggest that barred owls would need to be culled from approximately 40% of all occupied barred owl territories each year to reverse ongoing northern spotted owl population declines.

## INTRODUCTION

Estimating metrics associated with wildlife populations (e.g., density and occupancy) represents a cornerstone of wildlife research and provides critical information to guide management (Hone et al., 2010; Kéry, 2008). Information on population trends can help managers prioritize species for conservation action and assess viability of current practices in maintaining biodiversity (Barnes et al., 2016; Di Fonzo et al., 2016). Similarly, modeling minimum viable population size can aid in conservation planning and provide targets for recovery efforts (Flather et al., 2011; Traill et al., 2007). In the case of harvested animals, robust estimation of demographic parameters and population growth rates is frequently used to inform maximum sustainable yields (Buckland et al., 2000). Finally, relationships between environmental resource conditions and population metrics can also be used to assess the ability of conservation action to stabilize or reverse population declines (Crawford et al., 2018).

Efforts to enhance the estimation of populations and demographic parameters have long incorporated the influences one species can exert on another (Lotka, 1925; Volterra, 1928). Both ecological theory and empirical studies suggest that interspecific competition, facilitation, mutualism, parasitism, and predation can have dramatic influences on wildlife abundance (for example Bailey et al., 2009; Connell, 1983; MacArthur & Levins, 1967; MacKenzie et al., 2006; Svenning et al., 2014). As a result, potential influences of species co‐occurrence are increasingly incorporated in modeling efforts. For instance, competition for scarce resources can lower fitness directly by increasing the risk of predation (Halliday & Morris, 2013), reducing juvenile and adult survival (Manlick et al., 2021; Tanner, 1997), or limiting reproduction (Spagopoulou et al., 2020; Stockley & Bro‐Jørgensen, 2011). Species may facilitate the introduction of disease vectors, resulting in negative effects on a sympatric species (Crowl et al., 2008; Stephens et al., 2009). Additionally, species may enhance or diminish site suitability for other species (see Hirzel & Le Lay, 2008). Finally, and more recently, the influence of perceived predation on prey (i.e., “fear”) has been recognized and experimentally demonstrated (Lima, 1998; Zanette et al., 2011) with direct and transgenerational effects on prey populations (Allen et al., 2022; Guibert et al., 2013; Zanette et al., 2011).

Given the demonstrated effects species co‐occurrence have on wildlife populations, there is opportunity for models to incorporate these associated effects to improve population estimation procedures. Refined co‐occurrence models which better address effects of species co‐occurrence may lead to more robust estimation of appropriate harvest rates, invasibility of regions, reintroduction and translocation success rates, ecological response to predator control programs, invasive species control efficacy, and minimum viable populations. Collectively, these improvements can result in cascading economic, social, and ecological outcomes across ecosystems (Balmford & Bond, 2005; Epanchin‐Niell, 2017; Sala et al., 2000).

We incorporated species co‐occurrence effects in a modified Lotka–Volterra (hereafter, “L–V”) population model to evaluate if doing so can improve estimates of the proportion of occupied territories and provide more robust inference regarding ecological response to conservation action. We used the test case of northern spotted (*Strix occidentalis caurina*; hereafter, “NSO”) and barred (*Strix varia*; hereafter, “BAOW”) owls in the Pacific Northwest region of North America. Historically, the Pacific Northwest region supported only NSO populations; however, today BAOW occupancy surpasses NSO occupancy at sites where long‐term demographic data were collected (Franklin et al., 2021). This BAOW range expansion into the NSO range is believed to have been facilitated through forest fragmentation, changing climatic conditions, and afforestation of the Great Plains (Dark et al., 1998; Livezey, 2009). While these drivers are widely discussed in the literature, direct empirical evidence for their relative contributions remains limited, and some uncertainty persists (e.g., Wiens et al., 2014). Additionally, recent genetic analyses suggest that western BAOW populations may have diverged from eastern populations earlier than previously thought (Fujito et al., 2021), highlighting the complexity of historical range dynamics. NSO represent one of three distinct subspecies of spotted owl occurring in North America (Gutiérrez et al., 2020). They occur from southern British Columbia to northern California, primarily within 200 km of the Pacific coast (Gutiérrez et al., 2020). NSO became officially listed as threatened under the U.S. Endangered Species Act in 1990 (U.S. Department of the Interior, Federal Register, Part VI. Fish and Wildlife Service, 1990) due to declining population trends and survival rates primarily associated with habitat loss (U.S. Department of the Interior, Federal Register, Part VI. Fish and Wildlife Service, 1990).

A growing body of evidence indicates that BAOW may suppress NSO fitness through multiple mechanisms. First, observed interspecific agonistic interactions (Leskiw & Gutiérrez, 1998; Van Lanen et al., 2011) may result in direct NSO injury or mortality. Furthermore, NSO exhibit reduced calling rates and responsiveness in the presence of BAOW (Bailey et al., 2009; Crozier et al., 2006), largely attributed to interference competition (Crozier et al., 2006; Van Lanen et al., 2011), which may reduce the ability of NSO to defend territories and attract mates. There is also evidence of resource partitioning, with NSO using different habitats across a gradient of BAOW presence or territory overlap (Irwin et al., 2020; Wiens et al., 2014). Such resource partitioning in the presence of BAOW may result in NSO settling in suboptimal habitat, with cascading consequences to NSO reproduction and fitness (Wiens et al., 2014). These mechanisms, and potentially others yet untested, have resulted in declines of NSO occupancy rates and fitness in the presence of BAOW (Dugger et al., 2016; Franklin et al., 2021). These BAOW‐induced reductions in NSO fitness are so extreme that BAOW are now considered the primary factor affecting NSO demographic rates (Franklin et al., 2021). Other factors, including forest management, climate change, and large wildfires, may also contribute to local declines, though their range‐wide impacts appear limited compared to those of BAOW (Franklin et al., 2021). Indeed, two experimental studies have demonstrated that removal of BAOW can result in increased NSO demographic rates compared to control sites (Diller et al., 2016; Wiens et al., 2021). This negative effect of BAOW on NSO fitness is of critical concern to conservationists, given that NSO populations have continued to decline following their listing under the U.S. Endangered Species Act in 1990 (U.S. Department of the Interior, Federal Register, Part VI. Fish and Wildlife Service, 1990), with populations declining between 2% and 9% annually across monitored sites (Franklin et al., 2021). Apart from their effects on NSO, BAOW prey on a broad array of species, including endemics not taken by NSO, and their smaller home ranges combined with a diverse diet heighten the potential for shifts in community structure and trophic dynamics (Holm et al., 2016).

The magnitude to which BAOW are believed to suppress NSO populations is sufficiently large that managers have now finalized a Barred Owl Management Strategy, which provides guidance regarding the removal of wild BAOW from NSO habitat within the United States (i.e., culling) (U.S. Fish and Wildlife Service, 2024). This Barred Owl Management Strategy cites several supporting studies, which found evidence of competitive release for NSO when BAOW were experimentally removed from potential NSO habitat, including increased NSO occupancy, survival, and recruitment rates (Diller et al., 2016; Wiens et al., 2021). Furthermore, BAOW culling in the northern portion of the California Spotted Owl (hereafter; “CSO”; *Strix occidentalis occidentalis*) range resulted in CSO recolonizing regions they had formerly occupied (Hofstadter et al., 2022). Specifically, the Barred Owl Management Strategy provides recommendations for lethal removal of BAOW within the range of the NSO and CSO, with the goals of improving the survival and recovery of NSO and preventing declines in CSO due to BAOW competition (U.S. Fish and Wildlife Service, 2024). Recommendations provided within the strategy include prioritizing removal within NSO sites occupied in the last year; conducting BAOW removal at sites for a minimum of 5 years; concurrent monitoring for BAOW within removal areas; removing BAOW within multiple “general management areas” in each of 12 identified physiographic provinces (when possible) to maintain subpopulations; and only conducting removal on lands associated with willing landowners, land managers, and Tribes.

Recent advances in multi‐species population models, which account for competition, predation, facilitation, and fear effects (Grauer et al., 2025; MacKenzie et al., 2004; Rota et al., 2016), may enhance population estimates and yield more robust scientific inference. Although prior research has demonstrated depressed NSO occupancy rates and resource partitioning in the presence of BAOW (Dugger et al., 2011; Franklin et al., 2021; Kelly et al., 2003; Kroll et al., 2010; Olson et al., 2005; Wiens et al., 2014), to our knowledge, no efforts have been made to forecast how co‐occurrence effects may influence future NSO populations. Furthermore, we are unaware of efforts to simulate the potential influence culling may have on both NSO and BAOW populations, while accounting for co‐occurrence effects. Given the potential contentious nature of the proposed culling strategy, we feel it is prudent to develop the most robust models possible to evaluate the potential efficacy of such a program prior to its implementation. To address this information gap, we

1. Assessed the capability of a new population model, including co‐occurrence effects, to provide more robust population estimates of BAOW and NSO than previous models.
2. Evaluated if co‐occurrence effects are unidirectional (BAOW suppressing NSO) or bidirectional.
3. Simulated BAOW removal under the proposed culling program, using a population model including co‐occurrence effects, to re‐evaluate if culling BAOW may help conserve NSO.

Addressing these questions can help guide NSO management and inform potential BAOW culling programs in the Pacific Northwest. We believe the co‐occurrence model we evaluated has broad wildlife management applications including game management, predator control efforts, invasive species control, and translocation efforts. Our modeling effort is therefore transferrable to a broad suite of species, systems, and management challenges.

## METHODS

### Study system

NSOs are medium‐sized owls considered to be specialists of old‐growth coniferous forests (Gutiérrez et al., 2020). They primarily nest in tree cavities and forage on small mammals, including the dusky‐footed woodrat (*Neotoma fuscipes*) and northern flying squirrel (*Glaucomys sabrinus*) (Gutiérrez et al., 2020). They historically occurred from southern British Columbia, Canada, south through Pacific coastal forests to Marin County, California, United States (U.S. Dept. Interior, 1992). Today, NSOs occupy much of their historic range; however, the proportion of occupied sites has declined markedly, initially due to the harvest of mature coniferous forest for timber (Thomas et al., 1990) and later due to competition with BAOWs (Franklin et al., 2021). BAOWs are closely related to NSOs, and the two species have been known to interbreed infrequently (*n* = 47 out of over 9000 banded owls; Kelly & Forsman, 2004). BAOW and NSO hybridization results in offspring (“sparred owls”) characterized by plumage and vocalizations which are intermediate between the two species, but with body sizes exceeding those typically observed in BAOW or NSO (Hamer et al., 1994). Sparred owls are reproductively viable and produce offspring that can become difficult to identify when mated with BAOW or NSO (Kelly & Forsman, 2004).

BAOWs are largely considered generalists, exhibiting a broader diet and occupying home ranges, which are generally three to four times smaller than NSO (Gutiérrez et al., 2007; Hamer et al., 2007; Kryshak et al., 2022), which may also place pressure on other native species beyond NSO (Holm et al., 2016). Male and female BAOW body mass averages 5% and 22% more than NSO, respectively (Gutiérrez et al., 1995; Mazur & James, 2000). Collectively, the broader diet, smaller home range size, and larger body mass may contribute to BAOW behavioral dominance over and exclusion of NSO in areas of sympatry (Gutiérrez et al., 1995; Polis et al., 1989; Van Lanen et al., 2011). The historic range of BAOW did not overlap that of the NSO (Dark et al., 1998; Gutiérrez et al., 2007; Kelly et al., 2003; Livezey, 2009). The first BAOW specimen was taken in British Columbia, Canada in 1943 and the first offspring was reported in 1947 (Grant, 1966). From there, BAOW began extending their range southward, increasing overlap with the NSO range. In the United States, BAOW were first confirmed in Washington in 1965 (Rogers, 1977), Oregon in 1974 (Taylor & Forsman, 1976), and California in 1981 (Evens & LeValley, 1982). To date, the BAOW range is believed to completely overlap that of the NSO (Dark et al., 1998; Gutiérrez et al., 2007; Kelly et al., 2003; Livezey, 2009). Concurrent with BAOW range expansion, NSO population declines were documented across 11 long‐term demography study sites, resulting in the loss of >80% (Washington), >60% (Oregon), and >50% (California) of NSO individuals. As a result, BAOW are now considered the largest threat to continued NSO existence within their native range (Franklin et al., 2021). To address the BAOW threat to NSO, the USFWS has proposed a BAOW culling program to alleviate the potential extirpation of NSO by BAOW (U.S. Fish and Wildlife Service, 2024).

### Modeled data

**FIGURE 1:**
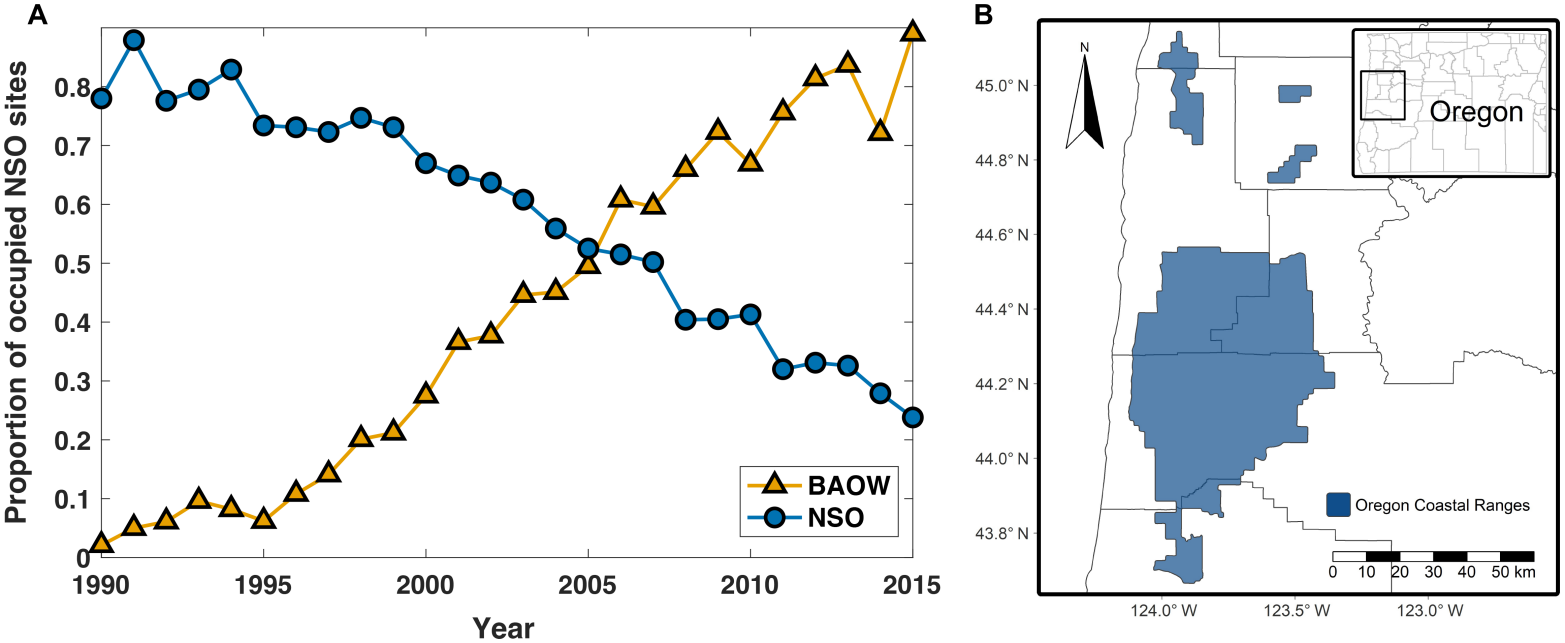
(A) Graphical representation of the proportion of northern spotted owl (NSO) sites inhabited by NSO (blue) and barred owl (orange, BAOW) within the Oregon Coast Range in the Pacific Northwest, USA. These data have been sourced from the 2016 annual USDA Forest Service report by Lesmeister et al. (2016). (B) The Oregon Coast Range study area in Oregon, USA, primarily located within public forest lands managed by the Siuslaw National Forest and the Bureau of Land Management's Salem and Eugene Districts.

We modeled NSO and BAOW populations as a function of co‐occurrence effects using raw data (Appendix S1: Table S1) from the 2016 annual USDA Forest Service report by Lesmeister et al. (2016). These data represented the proportion of NSO sites in which BAOW and NSO were detected from 1990 to 2015 within the Oregon Coast Range in the Pacific Northwest, USA (Figure 1A). These data were collected primarily on public lands managed by the USDA Forest Service (Figure 1B). From 1990 to 2015, the number of sites occupied by BAOWs increased from 3 to 124 (minimum = 3; maximum = 144). Conversely, the number of occupied sites by NSOs decreased from 110 to 48 (minimum = 48; maximum = 145) over the same timeframe. Owl occurrence data were collected using methods designed to elicit NSO responses (Lesmeister et al., 2016).

For model simplification, we assumed equal rates of NSO and BAOW detection. However, we acknowledge that detection rates among the two species have been shown to differ when sampling occurs using survey methodology akin to Lesmeister et al. (2016) (see Bailey et al., 2009). Bailey et al. (2009) further demonstrated the degree to which species‐specific detection rates differ likely varies as a function of survey conditions, time of day, the number of sampling visits, and true conspecific occupancy status at sites. Finally, we removed records of hybrid owls from the dataset (*n* = 18).

### Model formulation

#### L–V competition model

The classic L–V competition model (Equation 4) and its variants have been intensely investigated to study the population dynamics of two species that compete for limiting or scarce resources. These models estimate parameters such as growth rate and inter/intraspecific competition rate (Lou, 2008) and predict well‐observed biological states that include coexistence, competitive exclusion, and bi‐stability (Murray, 2007).

In the classic L–V formulation, the instantaneous growth rates (dx/dt and dy/dt) of two species (X and Y) are estimated as a cumulative effect of exploitation and interference competition (Begon et al., 1986). Mathematically, the L–V formulation can be expressed as:

(1) $$\left\{\begin{array}{l} \frac{\mathrm{dx}}{\mathrm{dt}}=x\left({\hat{r}}_{x}-p_{x}x\right)-q_{x}\mathrm{xy}\\ \frac{\mathrm{dy}}{\mathrm{dt}}=y\left({\hat{r}}_{y}-p_{y}y\right)-q_{y}\mathrm{xy}. \end{array}\right.$$

The terms ${\hat{r}}_{x}$ and ${\hat{r}}_{y}$ represent the intrinsic (per capita) growth rates. The intraspecific competition parameters ($p_{x}$ and $p_{y}$) and the interspecific competition parameters ($q_{x}$ and $q_{y}$) capture the combined effects of both exploitation and interference competition (refer to Appendix S1: Section S1 for details). Specifically, the intraspecific competition terms are given by $p_{x}={\bar{r}}_{x}+{\tilde{r}}_{x}$ for species x and $p_{y}={\bar{r}}_{y}+{\tilde{r}}_{y}$ for species y. Here, ${\bar{r}}_{x}$ and ${\bar{r}}_{y}$ represent the effects of intraspecific resource‐based (exploitation) competition, and ${\tilde{r}}_{x}$ and ${\tilde{r}}_{y}$ represent the effects of interspecific interference competition. Similarly, the interspecific competition terms are given by $q_{x}={\bar{b}}_{x}+{\tilde{b}}_{x}$ for species x and $q_{y}={\bar{b}}_{y}+{\tilde{b}}_{y}$ for species y. Here, ${\bar{b}}_{x}$ and ${\bar{b}}_{y}$ denote the interspecific competition rates due to exploitation, while ${\tilde{b}}_{x}$ and ${\tilde{b}}_{y}$ capture the effects of interspecific interference competition between species. Mathematically, this formulation can be expressed as:

(2) $$\left\{\begin{array}{l} \frac{\mathrm{dx}}{\mathrm{dt}}=\overset{\text{Growth}\;\text{term}\;\text{and}\;\text{exploitation}\;\text{competition}}{\overbrace{x\left({\hat{r}}_{x}-{\bar{r}}_{x}x-{\bar{b}}_{x}y\right)}}\;+\;\overset{\text{Interference}\;\text{competition}}{\overbrace{x\left(-{\tilde{r}}_{x}x-{\tilde{b}}_{x}y\right)}}\\ \frac{\mathrm{dy}}{\mathrm{dt}}=\overset{\text{Growth}\;\text{term}\;\text{and}\;\text{exploitation}\;\text{competition}}{\overbrace{y\left({\hat{r}}_{y}-{\bar{r}}_{y}x-{\bar{b}}_{y}y\right)}}\;+\;\overset{\text{Interference}\;\text{competition}}{\overbrace{y\left(-{\tilde{r}}_{y}x-{\tilde{b}}_{y}y\right)}}. \end{array}\right.$$

Thus, a co‐occurrence effect would potentially increase or decrease the ${\hat{r}}_{x}$ and ${\hat{r}}_{y}$ terms through exploitative competition. On rearranging (Equation 2) (or simply substituting $p_{x},p_{y},q_{x}$ and $q_{y}$ in the classical L–V formulation; Equation 1), the formulation yields:

(3) $$\left\{\begin{array}{l} \frac{\mathrm{dx}}{\mathrm{dt}}=x\left({\hat{r}}_{x}-\left({\bar{r}}_{x}+{\tilde{r}}_{x}\right)x\right)-\left({\bar{b}}_{x}+{\tilde{b}}_{x}\right)\mathrm{xy}\\ \frac{\mathrm{dy}}{\mathrm{dt}}=y\left({\hat{r}}_{y}-\left({\bar{r}}_{y}+{\tilde{r}}_{y}\right)y\right)-\left({\bar{b}}_{y}+{\tilde{b}}_{y}\right)\mathrm{xy}. \end{array}\right.$$

One can then generalize the above model by relabeling the constants as follows: $a_{11}={\hat{r}}_{x},a_{12}={\bar{r}}_{x}+{\tilde{r}}_{x},a_{21}={\hat{r}}_{y},a_{22}={\bar{r}}_{y}+{\tilde{r}}_{y},b_{12}={\bar{b}}_{x}+{\tilde{b}}_{x},$ and $b_{22}={\bar{b}}_{y}+{\tilde{b}}_{y}.$ By applying standard dimensional analysis, we can simplify (Equation 3) and the parameters therein (that is, going from 6 parameters to 4), to obtain a classical two‐species L–V ordinary differential equation (hereafter, “ODE”) competition model (refer to Appendix S1: Equation S6 for details). This model integrates both exploitation and interference competition and is represented by the following equations (Murray, 2007):

(4) $$\left\{\begin{array}{l} \frac{\mathrm{dx}}{\mathrm{dt}}=a_{1}x\left(1-x\right)-b_{1}\mathrm{xy}\\ \frac{\mathrm{dy}}{\mathrm{dt}}=a_{2}y\left(1-y\right)-b_{2}\mathrm{xy}. \end{array}\right.$$

#### BAOW co‐occurrence effects on NSO

To evaluate co‐occurrence effects BAOW may have on NSO, we modified (Equation 4) as described by Wang et al. (2016) (Equation 5). In this model, the growth rate of the apprehensive competitor, denoted as x, is not fixed, but exhibits density‐dependent behavior. Specifically, the growth rate diminishes by a factor ≈1/1+ky, where k≥0 represents co‐occurrence effects on NSO by BAOW. Consequently, a higher density of the competitor y intensifies the co‐occurrence effects on x. Conversely, when k=0, species co‐occurrence does not influence growth rate and the classic L–V model (Equation 4) is recovered.

Being motivated by the classical model, Srivastava et al. (2023) considered the classical two‐group L–V competition model with only one competitor causing fear to the other competitor. The resulting ODE model is as follows:

(5) $$\left\{\begin{array}{l} \frac{\mathrm{dx}}{\mathrm{dt}}=a_{1}x\left(\frac{1}{1+\mathrm{ky}}-x\right)-b_{1}\mathrm{xy}\\ \frac{\mathrm{dy}}{\mathrm{dt}}=a_{2}y\left(1-y\right)-b_{2}\mathrm{xy}, \end{array}\right.$$

Note, the above model is completely deterministic and does not incorporate environmental noise or stochastic effects. Using this model, we can then identify the potential equilibrium states (Table 1), using procedures described in Appendix S1.

**TABLE 1:** Parametric restriction concerning all possible equilibrium states for a modified Lotka–Volterra model, which incorporates unidirectional co‐occurrence effects (Equation 5).

Classical case	k>0	Ecological interpretation
Mutual extinction; ${\hat{E}}_{0}=\left(0,0\right)$	Locally unstable	Extinction of both species is not ecologically possible
NSO extinction; ${\hat{E}}_{1}=\left(0,1\right)$	Locally stable if $a_{1}<b_{1}\left(1+k\right)$	NSO growth rates are suppressed through competition with BAOW, resulting in local extinction of NSO
BAOW extinction; ${\hat{E}}_{2}=\left(1,0\right)$	Locally stable if $b_{2}>a_{2}$	Interspecific competition with NSO suppresses BAOW population growth rate and leads to local BAOW extinction
Coexistence; ${\hat{E}}_{3}=\left(x^{*},y^{*}\right)$	Locally stable if $a_{1}a_{2}-b_{1}b_{2}>{\mathrm{kb}}_{2}a_{1}$	The growth rates of both species surpass any interspecific co‐occurrence effects and both species are able to coexist in areas of sympatry

*Note*: The initial column enumerates equilibrium states, assuming an absence of co‐occurrence effects on northern spotted owl (NSO) by barred owl (BAOW). Conversely, the second and third columns list parametric restrictions and their ecological interpretations when co‐occurrence effects are considered. Detailed mathematical explanations are available in Appendix S1.

#### A case of the two‐way co‐occurrence effect

To construct a mathematical model in which both species influence the population of the other species, we altered (Equation 5) as shown in (Equation 6). Here, we denote the co‐occurrence effect of NSO on BAOW as m.

(6) $$\left\{\begin{array}{l} \frac{\mathrm{dx}}{\mathrm{dt}}=a_{1}x\left(\frac{1}{1+\mathrm{ky}}-x\right)-b_{1}\mathrm{xy}\\ \frac{\mathrm{dy}}{\mathrm{dt}}=a_{2}y\left(\frac{1}{1+\mathrm{mx}}-y\right)-b_{2}\mathrm{xy}, \end{array}\right.$$

#### Culling model

We evaluated the potential for culling to enhance NSO growth rates by introducing a term representing density‐dependent culling of BAOW $\left(c\right)$ in adapting (Equation 7). Here, c represents the proportion of NSO sites, occupied by BAOW, from which BAOW are entirely removed each year. Consequently, the resulting ODE can be written as

(7) $$\left\{\begin{array}{l} \frac{\mathrm{dx}}{\mathrm{dt}}=a_{1}x\left(\frac{1}{1+\mathrm{ky}}-x\right)-b_{1}\mathrm{xy}\\ \frac{\mathrm{dy}}{\mathrm{dt}}=a_{2}y\left(1-y\right)-b_{2}\mathrm{xy}-\mathrm{cy}, \end{array}\right.$$

#### Alternate culling model

We present a mathematical model (Equation 9) that builds upon (Equation 5) (Srivastava et al., 2023) by forecasting population predictions from 2016 to 2090 and incorporates a density‐dependent culling management strategy (denoted by parameter y) beginning in 2025. To formulate this model, we employed the indicator function $\chi_{\left[2025,\infty \right]}$, which yields a value of 1 when forecasting predictions and 0 for years 1990–2024. Mathematically, it is expressed as:

(8) $$\chi_{\left[2025,\infty \right]}=\left\{\begin{array}{l} 1,\;2025\;\text{onwards}\\ 0,\;\text{from}\;1990–2024. \end{array}\right.$$

Furthermore, we incorporate a density‐dependent culling factor affecting BAOW population densities (y). Consequently, we modify the traditional L–V equation to the following ODE model:

(9) $$\left\{\begin{array}{l} \frac{\mathrm{dx}}{\mathrm{dt}}=a_{1}x\left(\frac{1}{1+\mathrm{ky}}-x\right)-b_{1}\mathrm{xy}\\ \frac{\mathrm{dy}}{\mathrm{dt}}=a_{2}y\left(1-y\right)-b_{2}\mathrm{xy}-c\chi_{\left[2025,\infty \right]}y, \end{array}\right.$$

where $c\in \left[0,1\right]$ is the BAOW culling rate and $c\chi_{\left[2025,\infty \right]}$ represents the proportion of y culled per unit time after 2025.

### Modeling procedures

#### Nonlinear fitting and model selection techniques

Advanced algorithms to estimate parameters in nonlinear equations frequently require informed initialized values (Motulsky & Ransnas, 1987; Schoukens & Ljung, 2019). To address this, we used parameter estimates from recent NSO and BAOW studies as initial parameter values to help ensure that resulting model fit aligned with biological context and system dynamics.

To find the best‐fit parameters, we used the Bayesian approach, which is more appropriate because it better quantifies the uncertainty surrounding the estimates and avoids mixing analytical paradigms (Ghosh et al., 2007). We used the Metropolis–Hastings Markov chain Monte Carlo method for Bayesian parameter estimation (Chib, 2001). A uniform prior was assigned to each parameter within the interval $\left(0,10\right)$. This choice constrained the parameter space while remaining weakly informative within the specified range. The likelihood was based on Gaussian observational noise with a fixed SD of 0.05. To sample from the posterior distribution, we ran a single Markov chain Monte Carlo (MCMC) chain for 5000 iterations to ensure fine‐grained exploration of the parameter space, discarding the first 100 samples as burn‐in to enhance convergence. Parameter proposals were drawn from a Gaussian distribution centered at the current value, with a SD of 0.001 for each parameter. Posterior samples were used to compute the mean estimates for each parameter and the corresponding 95% Bayesian credible intervals (hereafter, “95% CrIs”). See the Python code in (https://doi.org/10.5066/P1P33RZS, EA_Code_Bay.py).

We conducted all model selection analyses in the Wolfram Mathematica software (Wolfram Research, Inc., 2024). Wolfram software first solves the corresponding ODEs by using the ParametricNDSolveValue function. This in‐built function generates a numerical solution of ODEs as a ParametricFunction object, which captures all system dynamics. Next, we implemented another in‐built function, Nonlinear ModelFit, on these ParametricFunctions to create a nonlinear model that best fits these provided data and creates a symbolic FittedModel object. Specifically, we used initial values of $a_{1}=0,a_{2}=1,b_{1}=0,b_{2}=0,$ and k=0.9. See the Mathematica code in (https://doi.org/10.5066/P1P33RZS, Review_CI_Bodine_Fear_Fit.nb).

#### Model evaluation

We evaluated our model using standard model selection methods including Likelihood ratio test: (Johnson & Omland, 2004; Sokal & Rohlf, 1995), corrected Akaike information criterion adjusted for small sample size (${\mathrm{AIC}}_{c}$; Burnham, 1998; White & Burnham, 1999), and Bayesian information criterion (BIC; Schwarz, 1978; Zucchini, 2000) (refer to Appendix S1: Table S3 for details). We used ${\mathrm{AIC}}_{c}$ to directly compare model performance of (Equation 5) and (Equation 6) to the model evaluated by Bodine and Capaldi (2017).

## RESULTS

### Model evaluation and parameter estimates

#### Comparison with Bodine and Capaldi (2017)

When comparing the Classical L–V model (Equation 4), one‐way co‐occurrence effects model (Equation 5) and two‐way co‐occurrence model (Equation 6), we found the one‐way co‐occurrence model resulted in the most parsimonious and robust fit (Appendix S1: Table S2). Our one‐way co‐occurrence model (Equation 5) carried 0.801 amount of the model weight of the three models evaluated. The two‐way co‐occurrence model (Equation 6) was the next best model, and carried 0.196 amount of the model weight. In contrast, the original L–V model explored by Bodine and Capaldi (2017) carried 0.003 amount of the model weight, indicating that it was not a competitive model in our model set (Appendix S1: Table S2).

#### Parameter fitting for the case BAOW co‐occurrence effects on NSO

We estimated the best‐fit parameters for our best‐fitting model (Equation 5) to these data with Bayesian 95% CrI (Table 2). We estimated the mean parameter, which indicates that the intrinsic growth $\left(a_{1}\right)$ and interspecific competition rates $\left(b_{1}\right)$ for NSO ($0.064\;\left[95\%\;\mathrm{CrI}\right]=(0.001,0.151)$ and $0.085\;\left[95\%\;\mathrm{CrI}\right]=(0.047,0.121)$, respectively) were lower than those estimated for BAOW ($0.547\;\left[95\%\;\mathrm{CrI}\right]=(0.492,0.585)$ and $0.307\;\left[95\%\;\mathrm{CrI}\right]=(0.248,0.345)$, respectively), whereas the co‐occurrence effect on NSO by BAOW $\left(k\right)$ is $4.725\;\left[95\%\;\mathrm{CrI}\right]=(4.685,4.771)$.

**TABLE 2:** Best‐fit parameters and associated statistical analyses for the one‐way co‐occurrence effect model (Equation 5) estimating northern spotted (NSO) and barred owl (BAOW) population growth rates along the Oregon Coast, USA 1990–2015.

Parameter	Description	Estimate	SE
$a_{1}$	Intrinsic growth rate for NSO	0.064	4.190 × 10^−4^
$b_{1}$	Interspecific competition rate for NSO	0.085	2.061 × 10^−4^
$a_{2}$	Intrinsic growth rate for BAOW	0.547	2.543 × 10^−4^
$b_{2}$	Interspecific competition rate for BAOW	0.307	2.696 × 10^−4^
k	Co‐occurrence effect on NSO by BAOW	4.725	2.430 × 10^−4^

**FIGURE 2:**
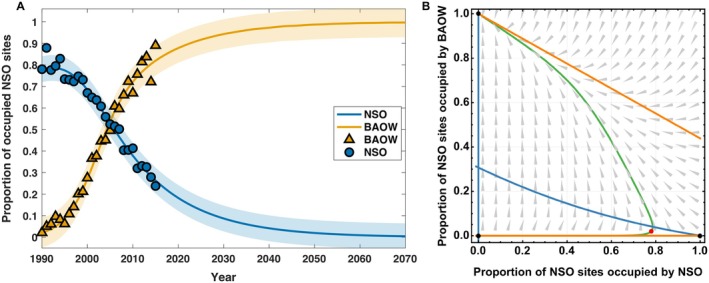
(A) Long‐time behavior of the one‐way co‐occurrence effects model (Equation 5), where orange triangles and blue circles enclosed within a black boundary indicate collected data points from Lesmeister et al. (2016) within the Oregon Coast Range in the Pacific Northwest, USA, while simulated lines represent the solution of model (Equation 5). The acronyms northern spotted owl (NSO) and barred owl (BAOW) are used for northern spotted and barred owls, respectively. The shaded areas represent the Bayesian credible interval of 95% CI. (B) Phase plots for one‐way co‐occurrence effects model (Equation 5) with optimized parameters display equilibrium points (black dots), x‐nullcline (blue curve), and y‐nullcline (orange curve). The red dot denotes initial proportion of NSO sites that are inhabited by NSO in 1990 within the Oregon Coast Range in the Pacific Northwest, USA, and the green curve traces the population trajectory converging to the NSO extinction state. (A phase plot is a geometric representation of trajectories for a given ordinary differential equation, starting from a point on a curve that represents an initial condition. A nullcline is a curve in the phase plane where the rate of change of a variable in an ordinary differential equation is zero.)

We used the above parameter estimates from our best‐fitting model to develop a long‐time dynamics plot (Figure 2A) and phase plot (Tabor, 1989; Figure 2B). The long‐time dynamics plot suggests that NSO are liable to become locally extinct by the year 2065. We note our estimate of time to NSO extinction is derived from our deterministic model, which may overestimate the time to NSO extinction (White, 2000). The phase plot (Figure 2B) suggests that there is no stable equilibrium where NSO and BAOW can coexist. Instead, all suitable NSO sites are expected to become occupied by BAOW.

### Reducing BAOW populations through culling

**FIGURE 3:**
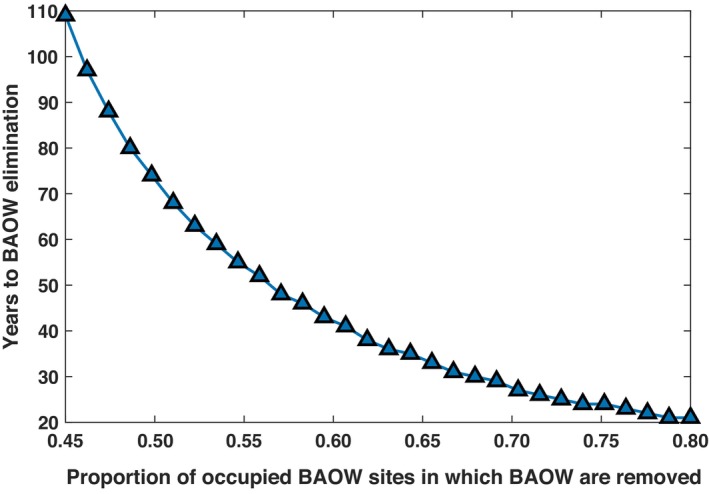
Estimated relationship between years to barred owl (BAOW) extirpation as a function of variable BAOW culling rates in the one‐way co‐occurrence effects model (Equation 5).

**FIGURE 4:**
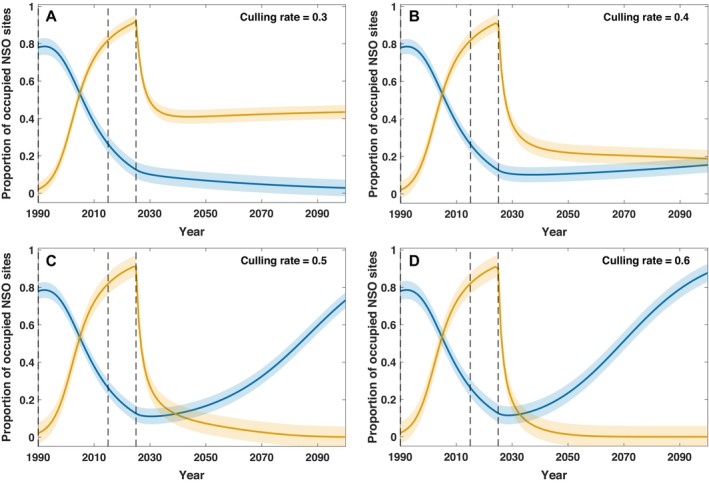
Long‐time behavior of the extended culling model (Equation 9), where blue and orange colored simulated lines represent the proportion of northern spotted owl (NSO) sites occupied by NSO and barred owl (BAOW), respectively, within the Oregon Coast Range in the Pacific Northwest, USA. In plot (A), culling rates are insufficient to maintain viable NSO populations and they become locally extinct. In contrast, culling rates of 0.4 (B) and larger (C and D) allow for continued NSO population persistence and result in more rapid extirpation of the BAOW from NSO sites.

**FIGURE 5:**
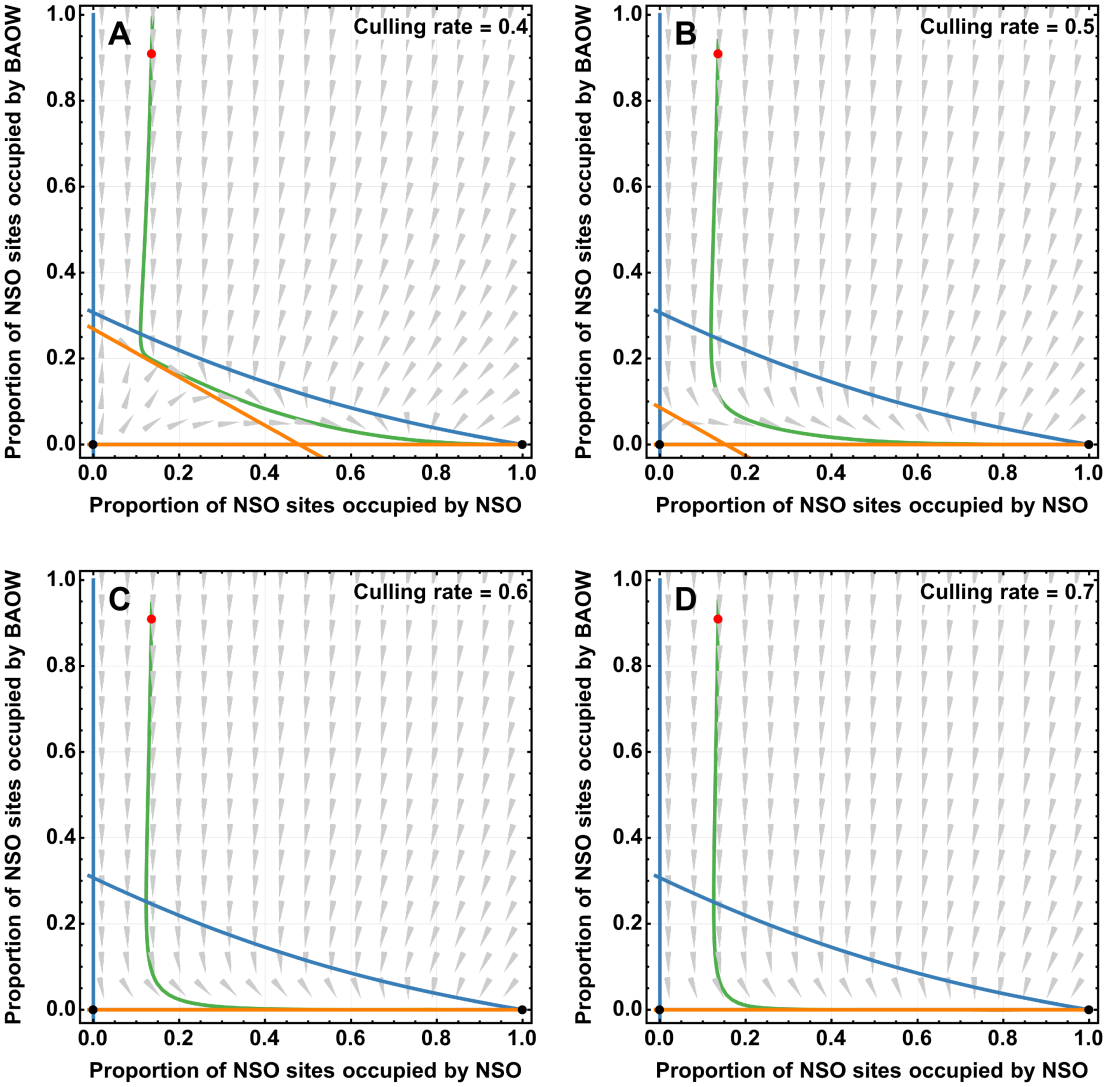
Phase plots for culling model (Equation 7) with optimized parameters display equilibrium points (black dots), x‐nullcline (blue curve), and y‐nullcline (orange curve). The red dot denotes initial proportion of northern spotted owl (NSO) sites that are inhabited by NSO in 2025 within the Oregon Coast Range in the Pacific Northwest, USA, and the green curve traces the population trajectory. The acronym BAOW is used for barred owls. (A phase plot is a geometric representation of trajectories for a given ordinary differential equation, starting from a point on a curve that represents an initial condition. A nullcline is a curve in the phase plane where the rate of change of a variable in an ordinary differential equation is zero.)

Using our best‐fit parameters from the top‐performing model (Equation 5), we employed variable culling rates to maintain century‐long coexistence between NSO and BAOW and completely eradicate BAOW within the NSO range. Results from our one‐way co‐occurrence effects model with culling (Equation 9) indicate that BAOW could be extirpated from NSO sites with a minimum culling rate of 0.45; however, at this rate, extirpation would require 110 years. Our results further indicate that annual culling rates of 0.8 may result in BAOW extirpation from NSO sites in as little as 20 years (Figure 3). Our model predicted NSO extinction when culling rate is less than 0.4 (Figures 4A and 5A). For example, given a culling rate of c=0.3, our model suggests that NSO will go locally extinct by the year 2090 (Figure 4A). Furthermore, our model predicts a potential coexistence for at least the next 100 years with a culling rate of c=0.4, eventually leading to the extirpation of the BAOW from the NSO sites in the long term (Figures 4B and 5B). We examined the cumulative number of BAOW, which may need to be culled under these various culling rates (Appendix S1: Figure S1). We calculated these values by multiplying the proportion of NSO sites occupied by BAOW by 204 (the maximum number of sites surveyed within the study area), followed by cumulative summation. Additionally, we multiplied the number of NSO sites where BAOW would need to be removed under each culling scenario by 6 individuals, given evidence that a single NSO site may support 2–4 BAOW territories (Wiens et al., 2014) and assuming that each BAOW territory would support a pair of individuals.

## DISCUSSION

Robust and unbiased estimation of wildlife populations is critical to the management and conservation of wild animal populations. Here, we introduce a modified L–V equation, which in part accounts for co‐occurrence effects on species and results in improved model fit compared to prior modeling iterations informed by these same data (Bodine & Capaldi, 2017). Our model partially addresses the lack of theory integrated into the declining population paradigm, which has been previously lamented (Caughley, 1994). Specifically, we provide a mathematical model addressing co‐occurrence effects, which demonstrates drivers of a declining population, and for which mechanisms have been well demonstrated (e.g., niche partitioning: Wiens et al., 2014; fear effects: Zanette et al., 2011; disease transmission: Crowl et al., 2008; Stephens et al., 2009). We extended this model to incorporate bidirectional co‐occurrence effects; however, we found little support for this model in our case study, as indicated by the lower log‐likelihood value and higher number of parameters compared to the one‐way co‐occurrence effect model (Appendix S1: Table S2). Our best‐fitting model, which included unidirectional co‐occurrence effects (BAOW population density suppressing NSO population growth), predicted that NSO populations might remain viable when BAOW culling rates meet or exceed 40% of occupied territories, a lower culling rate than Bodine and Capaldi (2017) estimated (c=0.47 or 47%). Our model provides theoretical support which concurs with empirical studies demonstrating NSO population recovery, given BAOW culling (Diller et al., 2016; Wiens et al., 2021). Our best‐fitting model further indicates that BAOW removal from >50% of NSO territories may lead to rapid NSO recovery, with NSO outnumbering BAOW within 14 years from 2025. We recognize that there may be biological, social, and logistical constraints that could prohibit BAOW culling at such a high rate, which we address below.

An examination of the sensitivity results in Table 1 highlights how variation in key parameters shape the long‐term qualitative behavior of the system. In particular, equilibrium outcomes are most sensitive to the interspecific competition coefficients and the strength of the co‐occurrence effect (k). Small increases in k can shift the system from coexistence to species exclusion (Table 1), which underscores the potential for abrupt regime shifts. Although our model is deterministic, we obtained the parameter estimates within a Bayesian framework, and the posterior distributions capture uncertainty associated with each parameter. Notably, the 95% CrIs for k often overlap with stability boundaries. This means alternative parameter values within the credible range can lead to contrasting ecological dynamics. These findings indicate that while our central conclusions are supported under the most likely parameter estimates, they are not robust across the full range of plausible values. It highlights the importance of accounting for parameter uncertainty in systems prone to threshold effects and suggests that additional empirical effort to refine parameter estimates could substantially improve the reliability of model‐based ecological predictions.

Our extension of the L–V model partially relaxes several L–V modeling assumptions, enhancing its ecological realism. By partially separating exploitative competition from interference competition, we account for the reduction in population growth rates of co‐occurring species, unidirectionally or bidirectionally. This change to the original model recognizes that wildlife does not always find ample food, so this model prevents species from reaching the maximum intrinsic growth rate (Murray, 2007). Recent field reports provide strong evidence that genetic adaptations and inter‐breeding are negligible (Hanna et al., 2018; U.S. Fish and Wildlife Service, 2024), which further justifies the use of our L–V model. Since we are utilizing differential equation models, with deterministic solutions, we can relax the assumption of randomness of solution (Murray, 2007). We also assume that population dynamics do not vary with the spatial arrangement of individuals on the landscape or the age structure of the populations. However, this is a limitation, as there are ecological studies indicating that, in long‐lived avian species like raptors, both age structure and the arrangement of individuals can significantly impact reproduction and survival (Blas et al., 2009; Sergio et al., 2011). Inspired by the classical L–V model, we also assume that populations do not have spatial or age distributions contributing to their dynamics.

Several recent studies have experimentally demonstrated the direct influence “fear” can have on populations (Allen et al., 2022; Palmer et al., 2022; Wang et al., 2016). Here, we wish to highlight the distinction between our model and what some have termed the “fear effect.” Our model formulation does not isolate influences fear may have on subordinate individuals from other co‐occurrence effects. Instead, it includes a parameter representing the comprehensive influence one species may have on another, such as exploitative and interference competition (including fear). We see our extension of the L–V model as an advancement in both the mathematical modeling and theory underpinning the declining species paradigm while acknowledging that our effort has limitations. First, we assumed that detection rates of BAOW and NSO were equal, and therefore proportions of undetected NSO and BAOW were comparable. However, rates of BAOW detection in the Lesmeister et al. (2016) dataset were likely lower than detection rates for NSO, given that the employed surveying methods were designed to elicit responses from NSO. Lower BAOW detection rates could result in an underestimate of the proportion of BAOW‐occupied sites, and a subsequent upward bias in the estimated per capita co‐occurrence effect of BAOW on NSO. This upward bias of the effect of BAOW on NSO may have caused our model to overestimate the proportion of BAOW sites that would need to be culled to facilitate NSO persistence. Additionally, we assumed that the index representing the proportion of occupied owl sites was representative of true site occupancy of the two species. However, prior work indicates that NSO responsiveness may decline with increasing BAOW presence (Crozier et al., 2006). Thus, the number of occupied NSO sites observed may have been less than the true number of NSO‐occupied sites, particularly in the latter years of the Lesmeister et al. (2016) dataset—when BAOW populations were relatively large. This again could result in an overestimation of the co‐occurrence effect of BAOW on NSO and an upward bias in the required BAOW culling rate for NSO persistence. Given these two sources of potential bias, the culling rate we estimated would be required to facilitate NSO persistence may be biased upwards and a lower rate of BAOW culling could potentially result in NSO recovery. Furthermore, the proportion of occupied NSO sites represents an index of BAOW and NSO presence, which may poorly relate to population estimates. For instance, BAOW have smaller home ranges than NSO and, therefore may occur at higher densities (Hamer et al., 2007; Wiens et al., 2014). Therefore, removing 40% of the BAOW population may be insufficient to reach the culling rate we estimated as necessary for NSO persistence (i.e., removal of BAOW from 40% of occupied NSO territories). Additionally, our investigation spanned a single population in Oregon and therefore does not include metapopulation structure. Therefore, our results may not be representative of range‐wide population outcomes and evaluation of a co‐occurrence effects model across the range of NSO to assess the potential efficacy of a range‐wide culling program may be warranted. We feel this is particularly important given that BAOW juveniles have been observed to disperse across distances exceeding 100 km (Watson et al., 2024), suggesting that population “rescue” from other populations is likely. Furthermore, our model is not spatially explicit and our estimates are based upon population‐level responses. In a spatially explicit model, culling could occur at local scales, reducing BAOW effects where it occurs (Wiens et al., 2021). However, in the ODE case, we consider culling will reduce the rangewide BAOW population, which serves as a useful threshold for conservation action.

Despite these limitations, we see our extension of the L–V model as representing both an immediate improvement (as indicated by model fit statistics) over prior large‐scale population models and a building block for increasingly sophisticated multi‐species population models, which better address biological and social realism. First, the model could readily be adapted to allow the strength of intraspecific competition among the subordinate species to vary as a density‐dependent process based upon the dominant species population. Such a model formulation would evaluate whether the subordinate species increasingly defends its territory against conspecifics to avoid occupying sites where the dominant species is present or which are otherwise unsuitable. We also note that our efforts did not incorporate refugia for the subordinate species, which we omitted from our model for two primary reasons. First, trends in the proportion of NSO territories occupied by BAOW rapidly approached 1 in our sample dataset (Lesmeister et al., 2016) and a recent meta‐analysis of a comprehensive NSO and BAOW dataset found that landscape habitat components could decrease negative effects of BAOW on NSO, but did not reverse negative population trends (Franklin et al., 2021). Together, these data and analyses suggest that there are few (if any) NSO territories unsuitable for BAOW. Second, BAOW natural history demonstrates that they act as habitat generalists, occupying a wide variety of mature forest communities and structures (Mazur & James, 2021). Thus, we felt that it was unlikely that BAOW would fail to occupy sites which are suitable for NSO. However, we acknowledge that the inclusion of a refugia parameter in a modified co‐occurrence L–V model may be appropriate for some systems. For instance, in the Hawaiin Islands, avian malaria (*Plasmodium relictum*) is spread by a mosquito (*Culex quinquefasciatus*), ultimately resulting in the mortality of Hawaiian honeycreepers (subfamily *Drepanidinae*). However, at higher elevations, temperatures become too cool for *C. quinquefasciatus*, providing refugia for the honeycreepers (Atkinson & LaPointe, 2009). In such systems, incorporation of the refugia parameter may be appropriate and result in more robust population estimates.

Another limitation of our study was that we estimated a static co‐occurrence parameter; however, the strength of co‐occurrence effects are liable to vary based on resource availability, resource preference by dominant and subordinate species across space, age structure and sex ratios (particularly when there is sexual dimorphism) of populations, and the proportion of paired individuals on the landscape. Future investigations into the strength of co‐occurrence effects across different systems, time, and space could provide new insights into the variability of any co‐occurrence effects. For instance, augmented models could evaluate the seasonality of co‐occurrence effects, potentially enhancing efficacy of conservation efforts. As an example, culling BAOW from historic NSO territories immediately prior to territory formation in the spring may better achieve desired NSO population targets, by reducing BAOW populations immediately prior to reproduction, compared to culling during different seasons. Similarly, over long timeframes, behavioral plasticity, as in the case of niche partitioning or the development of a search image, may alleviate or magnify co‐occurrence effects as behaviors are learned by modeled species over years and generations. At small scales, the competitive coefficient (k in Equation 5) may also vary based on the degree of home‐range overlap between the dominant and subordinate individuals. Thus, spatial modeling may lead to important advances regarding the spatial variability associated with co‐occurrence effects, and how such concepts can enhance management decision‐making.

Finally, future population modeling efforts which include a culling component could incorporate dynamic culling rates to reflect instances where culling rates are liable to decline as the culled population decreases. We see several potential biological, logistical, and social mechanisms, which may motivate the inclusion of density‐dependent culling rates in population modeling. First, foraging theory suggests that success rates of culling efforts may decline due to declining populations of the prey (i.e., the culled species; Brown et al., 1999). In our instance, after initial culling efforts, BAOW may find refugia in areas where the terrain makes access inconvenient or unsafe, lowering harvest rates. Additionally, the culled species may become wary over time, further reducing culling success rates. Finally, social considerations including personal ethics of would‐be hunters and hunting success rates could influence the level of culling that is attainable. For example, hunter motivation to cull may decline as hunting success rates decline and/or potentially as opposition to the act of culling native species increases (Kahlert et al., 2015; Woinarski, 2019). Accounting for these biological and logistical mechanisms influencing culling rates may represent important considerations when evaluating cost‐effectiveness of large‐scale culling efforts. Alternative management strategies, such as translocation or reproductive disruption through chemical or laparoscopic sterilization, are ineffective or infeasible for mitigating the threat posed by BAOW. There are no suitable relocation sites for the thousands of owls requiring removal, and sterilization methods are untested (Klug et al., 2023; Massei, 2023), expensive, and fail to address the immediate impact of adult BAOW on spotted owls. Moreover, BAOW has been found to occupy all NSO‐suitable habitats, preventing forest management from converting shared habitats into NSO‐only areas. Therefore, alternative strategies are currently neither viable nor scalable enough to reduce the threat posed by BAOW.

Our use of an ongoing and controversial case study demonstrates the utility of improved population estimation and the relevance of co‐occurrence effects for land and wildlife management. In our case study, we used our co‐occurrence model to explore species response to a culling program analogous to a predator control program. Yet, our model could also be used for many instances where management is designed to introduce, suppress, or divert populations, as in the cases of predator reintroductions, invasive species control, setting harvest limits, and human disturbance effects on sensitive wildlife. For many of these applications, assessing the relative strength of co‐occurrence effects could provide insights into optimizing management across density gradients of co‐occurring species (i.e., targeting areas supporting low densities of the dominant species when co‐occurrence effects on the subordinate species are large and vice versa). Finally, we wish to clarify that our efforts herein stemmed from our interest in improving population estimation procedures for the conservation of wildlife. We in no way are advocating for the culling of BAOW or other invasive species. Instead, our effort was intended to provide the best science available so managers and decision‐makers can make the most informed decision possible.

## AUTHOR CONTRIBUTIONS

Vaibhava Srivastava, Nicholas J. Van Lanen, and Rana D. Parshad conceptualized the idea for the study. Vaibhava Srivastava ran the simulations and completed the analysis with assistance from Rana D. Parshad. Nicholas J. Van Lanen and Vaibhava Srivastava wrote the manuscript with assistance from Rana D. Parshad. All authors gave their final approval for publication.

## CONFLICT OF INTEREST STATEMENT

The authors declare no conflicts of interest.

## Supporting information


Appendix S1.

## Data Availability

Data and code (Srivastava et al., 2025) are available via a U.S. Geological Survey data release in the USGS ScienceBase repository at https://doi.org/10.5066/P1P33RZS. Data were sourced from the 2015 annual USDA Forest Service report by Lesmeister et al. (2016).
