# Supplementary material for: Modeling species co‐occurrence effects to inform invasive barred owl management and recovery of the northern spotted owl
Source: Ecol Appl. 2026 Mar 12;36(2):e70195. doi: 10.1002/eap.70195 (PMC12982647; doi:10.1002/eap.70195)
Supplement: Supplementary file 1 — Appendix S1. [file EAP-36-e70195-s001.pdf]

# Modeling species co-occurrence effects to inform invasive barred owl management and recovery of the northern spotted owl

Vaibhava Srivastava[a], Nicholas J. Van Lanen[b], Rana D. Parshad[a,*]

[a]*Department of Mathematics, Iowa State University, Ames, IA 50011, USA*
[b]*US Geological Survey, Fort Collins Science Center, Fort Collins, CO 80526, USA*

---

Submitted to Ecological Applications

---

# Appendix S1

## Section S1: Derivation of the Lotka–Volterra competition model

The classical two-species Lotka–Volterra competition model (Begon et al., 1986; Jensen, 1987; Murray, 2007) is given by:

$$\begin{aligned}\frac{dx}{dt} &= \hat{r}_x x\Big(1 - \frac{x}{K_1}\Big) - q_y xy \\ \frac{dy}{dt} &= \hat{r}_y y\Big(1 - \frac{y}{K_2}\Big) - q_y xy\end{aligned} \tag{Eq S1}$$

Let's introduce the following change of variables: $p_x = \frac{\hat{r}_x}{K_1}$ and $p_y = \frac{\hat{r}_y}{K_2}$. Hence, (Eq S1) is transformed into the following system

*Corresponding author: Rana D. Parshad (rparshad@iastate.edu)
*Email addresses:* `vaibhava@iastate.edu` (Vaibhava Srivastava[a]), `nvan-lanen@fcgov.com` (Nicholas J. Van Lanen[b]), `rparshad@iastate.edu` (Rana D. Parshad[a,*])

$$\frac{dx}{dt} = x\Big(\hat{r}_x - p_x x\Big) - q_x xy$$
$$\frac{dy}{dt} = y\Big(\hat{r}_y - p_y y\Big) - q_y xy \qquad \text{(Eq S2)}$$

To separate the exploitation and interference competitions, let's introduce the following variables:

$$p_x = \bar{r}_x + \tilde{r}_x \quad \text{and} \quad p_y = \bar{r}_y + \tilde{r}_y$$
$$q_x = \bar{b}_x + \tilde{b}_x \quad \text{and} \quad q_y = \bar{b}_y + \tilde{b}_y. \qquad \text{(Eq S3)}$$

Specifically, the intraspecific competition terms are given by $p_x = \bar{r}_x + \tilde{r}_x$ for species $x$ and $p_y = \bar{r}_y + \tilde{r}_y$ for species $y$. Here, $\bar{r}_x$ and $\bar{r}_y$ represent the effects of intraspecific resource-based (exploitation) competition, and $\tilde{r}_x$ and $\tilde{r}_y$ represent the effects of interspecific interference competition. Similarly, $\bar{b}_x$ and $\bar{b}_y$ denote the interspecific competition rates due to exploitation, while $\tilde{b}_x$ and $\tilde{b}_y$ capture the effects of interspecific interference competition between species. Mathematically, the above Lotka–Volterra competition model can be expressed as:

$$\begin{aligned} \frac{dx}{dt} &= x\Big(\hat{r}_x - (\bar{r}_x + \tilde{r}_x)x\Big) - (\bar{b}_x + \tilde{b}_x)xy \\ \frac{dy}{dt} &= y\Big(\hat{r}_y - (\bar{r}_y + \tilde{r}_y)y\Big) - (\bar{b}_y + \tilde{b}_y)xy \end{aligned} \Rightarrow \begin{cases} \dfrac{dx}{dt} = \overbrace{x\left(\hat{r}_x - \bar{r}_x x - \bar{b}_x y\right)}^{\text{Growth term and exploitation competition}} + \overbrace{x\left(-\tilde{r}_x x - \tilde{b}_x y\right)}^{\text{Interference competition}} \\ \dfrac{dy}{dt} = \overbrace{y\left(\hat{r}_y - \bar{r}_y x - \bar{b}_y y\right)}^{\text{Growth term and exploitation competition}} + \overbrace{y\left(-\tilde{r}_y x - \tilde{b}_y y\right)}^{\text{Interference competition}}. \end{cases} \qquad \text{(Eq S4)}$$

One can then generalize the above model by relabeling the constants as follows:

$$a_{11} = \hat{r}_x \quad a_{12} = \bar{r}_x + \tilde{r}_x \quad a_{21} = \hat{r}_y \quad a_{22} = \bar{r}_y + \tilde{r}_y \quad b_1 = \bar{b}_x + \tilde{b}_x \quad b_2 = \bar{b}_y + \tilde{b}_y. \qquad \text{(Eq S5)}$$

By applying standard dimensional analysis, we can simplify, by reducing the parameters therein (that

is, going from 6 parameters to 4), to obtain a classical two-species L-V ordinary differential equation (hereafter, "ODE") competition model. On using the following change of variables: $x = \frac{a_{11}}{a_{12}}X, y = \frac{a_{21}}{a_{22}}Y$ and $t = \tau$, reduce the ODE from 6 parameters to 4 by substitution the above variables and using $\frac{dx}{dt} = K_x\frac{dX}{d\tau}$ and $\frac{dy}{dt} = K_y\frac{dY}{d\tau}$. Hence, we have

$$\begin{array}{l} \cancel{K_x}\frac{dX}{d\tau} = \cancel{K_x}X(a_{11} - a_{11}X) - b_2\cancel{K_x}K_yXY \implies \frac{dX}{d\tau} = a_1X(1-X) - b_1XY \\ \cancel{K_y}\frac{dY}{d\tau} = K_yY(a_{21} - a_{21}Y) - b_2K_x\cancel{K_y}XY \implies \frac{dY}{d\tau} = a_2Y(1-Y) - b_2XY \end{array}. \quad \text{(Eq S6)}$$

On again, relabeling the variables $X, Y$ and $\tau$, as $x, y$ and $t$, we have a model integrates both exploitation and interference competition and is represented by the following equations (Murray [2007]):

$$\begin{cases} \dfrac{dx}{dt} = a_1x\Big(1-x\Big) - b_1xy \\ \dfrac{dy}{dt} = a_2y\Big(1-y\Big) - b_2xy. \end{cases} \quad \text{(Eq S7)}$$

## Section S2: Sensitivity analysis

### Section S2.1: Sensitivity analysis for the classical two-species Lotka-Volterra (L-V) model

***Existence***

The nullclines associated with (Eq. 4) are:

$$x\Big(a_1 - a_1x - b_1y\Big) \quad \text{and} \quad y\Big(a_2 - a_2y - b_2x\Big). \quad \text{(Eq S8)}$$

Hence, the boundary equilibrium points are obtained by substituting $x = 0$ and $y = 0$ in the above equations of the nullclines, respectively. Denote the equilibrium points:

1. Mutual extinction: $\widehat{\mathbf{E}}_{\mathbf{0}} = (0, 0)$.
2. Northern spotted owl (hereafter, "NSO") extinction: $\widehat{\mathbf{E}}_{\mathbf{1}} = (0, 1)$.
3. Barred owl (hereafter, "BAOW") extinction: $\widehat{\mathbf{E}}_{\mathbf{2}} = (1, 0)$.
4. Coexistence: $\widehat{\mathbf{E}}_{\mathbf{3}} = (x^*, y^*) = \Big(\dfrac{a_2(a_1 - b_1)}{a_1a_2 - b_1b_2}, \dfrac{a_1(a_2 - b_2)}{a_1a_2 - b_1b_2}\Big)$ provided $x^*, y^* > 0$.

***Linear stability analysis***

We next perform stability analysis on the equilibrium points of (Eq. 4). The Jacobian matrix of system (Eq. 4) is given by

$$\widehat{J}^*(x^*, y^*) = \begin{pmatrix} a_1 - 2a_1x^* - b_1y^* & -b_1x^* \\ -b_2y^* & a_2 - 2a_2y^* - b_2x^* \end{pmatrix}. \tag{Eq S9}$$

**Lemma S.1.** $\widehat{\mathbf{E}}_{\mathbf{0}} = (0, 0)$ is locally unstable.

*Proof.* On evaluating $\widehat{J}^*$ at $\widehat{\mathbf{E}}_{\mathbf{0}}$, we have

$$\widehat{J}^*(\widehat{\mathbf{E}}_{\mathbf{0}}) = \begin{pmatrix} a_1 & 0 \\ 0 & a_2 \end{pmatrix}. \tag{Eq S10}$$

Being a triangular matrix, we know the above matrix has two positive eigenvalues: $a_1$ and $a_2$. Hence, the equilibrium point $\widehat{\mathbf{E}}_{\mathbf{0}}$ is locally unstable. □

**Lemma S.2.** $\widehat{\mathbf{E}}_{\mathbf{1}} = (0, 1)$ is locally stable iff $b_1 > a_1$.

*Proof.* On evaluating $\widehat{J}^*$ at $\widehat{\mathbf{E}}_{\mathbf{1}}$, we obtain

$$\widehat{J}^*(\widehat{\mathbf{E}}_{\mathbf{1}}) = \begin{pmatrix} a_1 - b_1 & 0 \\ -b_2 & -a_2 \end{pmatrix}. \tag{Eq S11}$$

Being a triangular matrix, the above matrix has two eigenvalues, $\lambda_1 = a_1 - b_1$ and $\lambda_2 = -a_2$. As $\lambda_2$ is always negative, if we can show that $\lambda_1$ is negative, we are done. Under the assumption, $b_1 > a_1 \iff \lambda_1 < 0$. Therefore, the boundary equilibrium point $\widehat{\mathbf{E_1}}$ is locally stable. □

**Lemma S.3.** $\widehat{\mathbf{E_2}} = (1, 0)$ is locally stable if $b_2 > a_2$.

*Proof.* On evaluating $\widehat{J}^*$ at $\widehat{\mathbf{E_2}}$,we obtain

$$\widehat{J}^*(\widehat{\mathbf{E_2}}) = \begin{pmatrix} -a_1 & -b_1 \\ 0 & a_2 - b_2 \end{pmatrix}. \qquad \text{(Eq S12)}$$

Being a triangular matrix, the above matrix has two eigenvalues, $\lambda_1 = -a_1$ and $\lambda_2 = a_2 - b_2$. As $\lambda_1$ is always negative, if we can show that $\lambda_2$ is negative, we are done. Under the assumption, $b_2 > a_2$, which implies $\lambda_2 < 0$. Therefore, the boundary equilibrium point $\widehat{\mathbf{E_2}}$ is locally stable. □

**Lemma S.4.** The interior equilibrium $\widehat{\mathbf{E_3}}$ exists if $a_1 > b_1$ and $a_2 > b_2$, and is locally stable if $a_1 a_2 > b_1 b_2$

*Proof.* On evaluating $\widehat{J}^*$ at $\widehat{\mathbf{E_3}} = (x^*, y^*)$, we have

$$\widehat{J}^*(\widehat{\mathbf{E_3}}) = \begin{pmatrix} -a_1 x^* & -b_1 x^* \\ -b_2 y^* & -a_2 y^* \end{pmatrix}. \qquad \text{(Eq S13)}$$

For the local stability of $\widehat{\mathbf{E_3}}$, it is enough to show that $Trace(\widehat{J}^*(\widehat{\mathbf{E_3}})) < 0$ and $Det(\widehat{J}^*(\widehat{\mathbf{E_3}})) > 0$. Simple computations yield

$$Trace(\widehat{J}^*(\widehat{\mathbf{E_3}})) = -a_1 x^* - a_2 y^* < 0, \qquad \text{(Eq S14)}$$

and

$$Det(\widehat{J}^*(\widehat{\mathbf{E}}_{\mathbf{3}}) = x^*y^*\Big\{a_1a_2 - b_2b_1\Big\}. \quad \text{(Eq S15)}$$

Under the assumption,

$$a_1a_2 > b_1b_2 \implies Det(\widehat{J}^*(\widehat{\mathbf{E}}_{\mathbf{3}})) > 0 \quad \text{(Eq S16)}$$

and the result follows.

□

**Section S2.2: Sensitivity analysis for the case BAOW co-occurrence effects on NSO**

***Existence***

The nullclines associated with (Eq. 5) are:

$$x\Big(\frac{a_1}{1+ky} - a_1x - b_1y\Big) \quad \text{and} \quad y\Big(a_2 - a_2y - b_2x\Big). \quad \text{(Eq S17)}$$

Hence, the boundary equilibrium points are obtained by substituting $x = 0$ and $y = 0$ in the above equations of the nullclines, respectively. Denote the equilibrium points:

1. Mutual extinction: $\widehat{\mathbf{E}}_{\mathbf{0}} = (0, 0)$.
2. NSO extinction: $\widehat{\mathbf{E}}_{\mathbf{1}} = (0, 1)$.
3. BAOW extinction: $\widehat{\mathbf{E}}_{\mathbf{2}} = (1, 0)$.

4. Co-existence: $\widehat{\mathbf{E}}_\mathbf{3}$ if $(x^*, y^* > 0)$

$$\widehat{\mathbf{E}}_\mathbf{3} = \left(x_i^*, y_i^*\right)_{i=1,2} = \Big(\frac{a_2(-(1+k)a_1a_2 + (1+2k)b_1b_2 \pm \Delta)}{2b_2k\,(b_1b_2 - a_1a_2)}, \frac{(-1+k)a_1a_2 + b_1b_2 \pm \Delta}{2k(a_1a_2 - b_1b_2)}\Big), \tag{Eq S18}$$

where $\Delta = \sqrt{(a_1a_2(k-1) + b_1b_2)^2 + 4a_1k\,(a_2 - b_2)\,(a_1a_2 - b_1b_2)}$.

***Linear stability analysis***

We next perform stability analysis on the equilibrium points of (Eq. 5). The Jacobian matrix of system (Eq. 5) is given by

$$\widehat{J}^*(x^*, y^*) = \begin{pmatrix} \dfrac{a_1}{1+ky^*} - 2a_1x^* - b_1y^* & \dfrac{-a_1kx^*}{(1+ky^*)^2} - b_1x^* \\ -b_2y^* & a_2 - 2a_2y^* - b_2x^* \end{pmatrix}. \tag{Eq S19}$$

**Lemma S.5.** $\widehat{\mathbf{E}}_\mathbf{0} = (0,0)$ is locally unstable.

*Proof.* On evaluating $\widehat{J}^*$ at $\widehat{\mathbf{E}}_\mathbf{0}$, we have

$$\widehat{J}^*(\widehat{\mathbf{E}}_\mathbf{0}) = \begin{pmatrix} a_1 & 0 \\ 0 & a_2 \end{pmatrix}. \tag{Eq S20}$$

Being a triangular matrix, we know the above matrix has two positive eigenvalues: $a_1$ and $a_2$. Hence, the equilibrium point $\widehat{\mathbf{E}}_\mathbf{0}$ is locally unstable. □

**Lemma S.6.** $\widehat{\mathbf{E}}_\mathbf{1} = (0,1)$ is locally stable iff $\dfrac{a_1}{1+k} < b_1$.

*Proof.* On evaluating $\widehat{J}^*$ at $\widehat{\mathbf{E}}_\mathbf{1}$, we obtain

$$\widehat{J}^*(\widehat{\mathbf{E}}_\mathbf{1}) = \begin{pmatrix} \dfrac{a_1}{1+k} - b_1 & 0 \\ -b_2 & -a_2 \end{pmatrix}. \qquad \text{(Eq S21)}$$

Being a triangular matrix, the above matrix has two eigenvalues, $\lambda_1 = \dfrac{a_1}{1+k} - b_1$ and $\lambda_2 = -a_2$. As $\lambda_2$ is always negative, if we can show that $\lambda_1$ is negative, we are done. Under the assumption,

$$\frac{a_1}{1+k} < b_1 \iff \lambda_1 < 0. \qquad \text{(Eq S22)}$$

Therefore, the boundary equilibrium point $\widehat{\mathbf{E}}_\mathbf{1}$ is locally stable. □

**Lemma S.7.** $\widehat{\mathbf{E}}_\mathbf{2} = (1, 0)$ is locally stable if $b_2 > a_2$.

*Proof.* On evaluating $\widehat{J}^*$ at $\widehat{\mathbf{E}}_\mathbf{2}$, we obtain

$$\widehat{J}^*(\widehat{\mathbf{E}}_\mathbf{2}) = \begin{pmatrix} -a_1 & -a_1 k - b_1 \\ 0 & a_2 - b_2 \end{pmatrix}. \qquad \text{(Eq S23)}$$

Being a triangular matrix, the above matrix has two eigenvalues, $\lambda_1 = -a_1$ and $\lambda_2 = a_2 - b_2$. As $\lambda_1$ is always negative, if we can show that $\lambda_2$ is negative, we are done. Under the assumption, $b_2 > a_2$, which implies $\lambda_2 < 0$. Therefore, the boundary equilibrium point $\widehat{\mathbf{E}}_\mathbf{2}$ is locally stable. □

**Lemma S.8.** The interior equilibrium $\widehat{\mathbf{E}}_\mathbf{3}$ exists and is locally stable if $\dfrac{a_1 a_2 - b_1 b_2}{b_2 a_1} > k$.

*Proof.* On evaluating $\widehat{J}^*$ at $\widehat{\mathbf{E}}_\mathbf{3} = (x^*, y^*)$, we have

$$\widehat{J}^*(\widehat{\mathbf{E}}_\mathbf{3}) = \begin{pmatrix} -a_1x^* & \dfrac{-a_1kx^*}{(1+ky^*)^2} - b_1x^* \\ -b_2y^* & -a_2y^* \end{pmatrix}. \qquad \text{(Eq S24)}$$

For the local stability of $\widehat{\mathbf{E}}_\mathbf{3}$, it is enough to show that $Trace(\widehat{J}^*(\widehat{\mathbf{E}}_\mathbf{3})) < 0$ and $Det(\widehat{J}^*(\widehat{\mathbf{E}}_\mathbf{3})) > 0$. Simple computations yield $Trace(\widehat{J}^*(\widehat{\mathbf{E}}_\mathbf{3})) = -a_1x^* - a_2y^* < 0$, and

$$Det(\widehat{J}^*(\widehat{\mathbf{E}}_\mathbf{3}) = x^*y^*\Big\{a_1a_2 - b_2\Big(\frac{a_1k}{(1+ky^*)^2} + b_1\Big)\Big\}. \qquad \text{(Eq S25)}$$

Note that $a_1k > \frac{a_1k}{(1+kx^*)^2}$. Therefore, if $k$ is chosen s.t, $\frac{a_1a_2-b_1b_2}{b_2a_1} > k$, then, $a_1a_2 > kb_2a_1 + b_1b_2$, which implies $Det(\widehat{J}^*(\widehat{\mathbf{E}}_\mathbf{3})) > 0$, and the result follows.

□

**Section S2.3: Sensitivity analysis for the case of the two-way co-occurrence effect**

***Existence***

The dynamical analysis of (Eq. 6) when $k, m > 0$ leads to $4^{th}$ order polynomial analysis. The (Eq. 6) possesses the following biologically feasible equilibria. These are

1. Mutual extinction: $\widehat{\mathbf{E}}_\mathbf{0} = (0, 0)$.
2. NSO extinction: $\widehat{\mathbf{E}}_\mathbf{1} = (0, 1)$ .
3. BAOW extinction: $\widehat{\mathbf{E}}_\mathbf{2} = (1, 0)$ .
4. Co-existence: $\widehat{\mathbf{E}}_\mathbf{3} = (x_i^*, y_i^*)_{i=1,2,3,4}$.

where

$$x^* = \frac{1}{a_1}\left(\frac{a_1}{1+ky^*} - b_1 y^*\right) \quad \text{(Eq S26)}$$

and $y^*$ is a positive root of the following fourth-order polynomial

$$A(y^*)^4 + B(y^*)^3 + C(y^*)^2 + D(y^*) + E = 0 \quad \text{(Eq S27)}$$

where,

$$\begin{aligned}
A &= b_1 k^2 m\left(a_1 a_2 - b_1 b_2\right),\\
B &= -k\left(a_1 a_2 - b_1 b_2\right)\left(a_1 k - 2b_1 m\right),\\
C &= a_2 a_1\left(-a_1 k m - 2a_1 k + b_1 m\right) + b_1 b_2\left(2k\left(a_1 m + a_1\right) - b_1 m\right) + a_2 a_1^2 k^2,\\
D &= b_2\left(b_1\left(2a_1 m + a_1\right) - a_1 a_1 k\right) + 2a_2 a_1^2 k - a_2 a_1\left(a_1 m + a_1\right),\\
E &= a_2 a_1^2 - a_1 b_2\left(a_1 m + a_1\right).
\end{aligned} \quad \text{(Eq S28)}$$

***Linear stability analysis for the case of NSO extinction***

We next perform stability analysis on the equilibrium points of (Eq. 6). The Jacobian matrix of (Eq. 6) is given by

$$\widehat{J}^*(x^*, y^*) = \begin{pmatrix} \dfrac{a_1}{1+ky^*} - 2a_1 x^* - b_1 y^* & \dfrac{-a_1 k x^*}{(1+ky^*)^2} - b_1 x^* \\ \dfrac{-a_2 m y^*}{(1+mx^*)^2} - b_2 y^* & \dfrac{a_2}{1+mx^*} - 2a_2 y^* - b_2 x^* \end{pmatrix}. \quad \text{(Eq S29)}$$

**Lemma S.9.** $\widehat{\mathbf{E}}_{\mathbf{1}} = (0, 1)$ is locally stable iff $\dfrac{a_1}{1+k} < b_1$.

*Proof.* On evaluating $\widehat{J}^*$ at $\widehat{\mathbf{E}}_\mathbf{1}$ and obtain

$$\widehat{J}^*(\widehat{\mathbf{E}}_\mathbf{1}) = \begin{pmatrix} \dfrac{a_1}{1+k} - b_1 & 0 \\ -a_2 m - b_2 & -a_2 \end{pmatrix}. \tag{Eq S30}$$

Being a triangular matrix, the above matrix has two eigenvalues, $\lambda_1 = \dfrac{a_1}{1+k} - b_1$ and $\lambda_2 = -a_2$. As $\lambda_2$ is always negative, if we can show that $\lambda_1$ is negative, we are done. Under the assumption, $\frac{a_1}{1+k} < b_1 \iff \lambda_1 < 0$ Therefore, the boundary equilibrium point $\widehat{\mathbf{E}}_\mathbf{1}$ is locally stable. □

## Section S2.4: Sensitivity analysis for culling model

### *Existence*

The nullclines associated with (Eq. 7) are:

$$x\Big(\frac{a_1}{1+ky} - a_1 x - b_1 y\Big) \quad \text{and} \quad y\Big(a_2 - a_2 y - b_2 x\Big) - cy. \tag{Eq S31}$$

Hence, the boundary equilibrium points are obtained by substituting $x = 0$ and $y = 0$ in the above equations of the nullclines, respectively. Denote the boundary equilibrium points:

- Mutual extinction: $\widehat{\mathbf{E}}_\mathbf{0} = (0, 0)$.
- NSO extinction: $\widehat{\mathbf{E}}_\mathbf{1} = \Big(0, 1 - \dfrac{c}{a_2}\Big)$.
- BAOW extinction: $\widehat{\mathbf{E}}_\mathbf{2} = (1, 0)$.

- Co-existence: $\widehat{\mathbf{E}}_\mathbf{3}$ if $x^*, y^* > 0$

$$\widehat{\mathbf{E}}_\mathbf{3} = \left(x_i^*, y_i^*\right)_{i=1,2} = \left(\tfrac{ck(a_1a_2 - 2b_1b_2) + a_2(b_1b_2(2k+1) - a_1a_2(k+1)) \pm a_2\Delta}{2b_2k(b_1b_2 - a_1a_2)}, \tfrac{a_1(-c)k + a_2a_1k - a_2a_1 + b_1b_2 \pm \Delta}{2k(a_1a_2 - b_1b_2)}\right), \quad \text{(Eq S32)}$$

where $\Delta = \sqrt{(a_1(ck - a_2(k-1)) - b_1b_2)^2 - 4a_1k(a_1a_2 - b_1b_2)(-a_2 + b_2 + c)}$.

***Linear stability analysis***

We next perform stability analysis on the equilibrium points of system (Eq. 7). The Jacobian matrix of system (Eq. 7) is given by

$$\widehat{J}^*(x^*, y^*) = \begin{pmatrix} \dfrac{a_1}{ky^*+1} - 2a_1x^* - b_1y^* & -\dfrac{a_1kx^*}{(ky^*+1)^2} - b_1x^* \\ -b_2y^* & -2a_2y^* + a_2 - b_2x^* - c \end{pmatrix} \quad \text{(Eq S33)}$$

To perform a comprehensive stability analysis of the culling model (Eq. 4), we plan to improve precision and applicability by keeping the parameters $a_1$, $a_2$, $b_1$, $b_2$, and $k$ constant at their best-fit values. Our goal is to enhance our understanding of the model by investigating the impact of the parameter $c$ on its behavior, which we expect will provide detailed insights.

$$\widehat{J}^*(x^*, y^*) = \begin{pmatrix} -0.128x^* - 0.085y^* + \frac{0.064}{4.725y^*+1} & -\frac{0.302x^*}{(4.725y^*+1)^2} - 0.085x^* \\ -0.307y^* & -c - 0.307x^* - 1.094y^* + 0.547 \end{pmatrix} \quad \text{(Eq S34)}$$

The boundary equilibrium points with respect to the best-fit parameters are defined as

1. Mutual extinction: $\widehat{\mathbf{E}}_\mathbf{0} = (0, 0)$.

2. NSO extinction:$\widehat{\mathbf{E}}_{\mathbf{1}} = (0, 1 - 1.828c)$.

3. BAOW extinction: $\widehat{\mathbf{E}}_{\mathbf{2}} = (1, 0)$.

**Lemma S.10.** $\widehat{\mathbf{E}}_{\mathbf{0}} = (0, 0)$ is locally unstable.

*Proof.* On evaluating $\widehat{J}^*$ at $\widehat{\mathbf{E}}_{\mathbf{0}}$, we obtain

$$\widehat{J}^*(\widehat{\mathbf{E}}_{\mathbf{0}}) = \begin{pmatrix} 0.064 & 0 \\ 0 & 0.547 - c \end{pmatrix} \quad \text{(Eq S35)}$$

Being a triangular matrix, the above matrix has two eigenvalues, $\lambda_1 = 0.064$ and $\lambda_2 = 0.547 - c$. $\lambda_1$ is always positive independent of the choice of $c$. Hence, the equilibrium point $\widehat{\mathbf{E}}_{\mathbf{0}}$ is locally unstable. □

**Lemma S.11.** $\widehat{\mathbf{E}}_{\mathbf{1}} = (1, 0)$ is locally stable if $c > 0.24$.

*Proof.* On evaluating $\widehat{J}^*$ at $\widehat{\mathbf{E}}_{\mathbf{1}}$, we obtain

$$\widehat{J}^*(\widehat{\mathbf{E}}_{\mathbf{1}}) = \begin{pmatrix} -0.064 & -0.3874 \\ 0 & 0.24 - c \end{pmatrix}. \quad \text{(Eq S36)}$$

Being a triangular matrix, the above matrix has two eigenvalues, $\lambda_1 = -0.064$ and $\lambda_2 = 0.24 - c$. Under the assumption, $c > 0.24 \iff \lambda_2 < 0$. Therefore, the boundary equilibrium point $\widehat{\mathbf{E}}_{\mathbf{1}}$ is locally stable. □

**Lemma S.12.** $\widehat{\mathbf{E}}_{\mathbf{2}} = (0, 1 - 1.828c)$ is locally stable if $c < 0.0.386$.

*Proof.* On evaluating $\widehat{J}^*$ at $\widehat{\mathbf{E}}_{\mathbf{2}}$, we obtain

$$\widehat{J}^*(\widehat{\mathbf{E}}_{\mathbf{2}}) = \begin{pmatrix} 0.155c - \frac{0.007}{c-0.663} - 0.085 & 0 \\ 0.561c - 0.307 & c - 0.547 \end{pmatrix} \qquad \text{(Eq S37)}$$

We need

$$c - 0.547 < 0 \implies 0 < c < 0.547 \qquad \text{(Eq S38)}$$

For $\lambda_2 < 0$, we need to solve the other inequality:

$$\frac{0.007}{0.663 - c} + 0.155c - 0.085 < 0 \quad \implies \quad 0.663 < c < 0.826 \quad \& \quad c < 0.386. \qquad \text{(Eq S39)}$$

Hence, under the assumption $c < 0.386$, which implies $\lambda_1, \lambda_2 < 0$. Therefore, the boundary equilibrium point $\widehat{\mathbf{E}}_{\mathbf{2}}$ is locally stable. □

# References


Begon, M., Harper, J. L., Townsend, C. R., et al. (1986). *Ecology: Individuals, Populations and Communities.* Blackwell Scientific Publications.

Jensen, A. L. (1987). Simple Models for Exploitative and Interference Competition. *Ecological Modelling*, 35(1-2):113–121.

Johnson, J. B. and Omland, K. S. (2004). Model Selection in Ecology and Evolution. *Trends in Ecology & Evolution*, 19(2):101–108.

Lesmeister, D., McCafferty, C., Beerweiler, K., Kumar, S., Lerro, N., Mason, B., Meiering, B., Skybak, K., Thomas, A., and Wert, K. (2016). Demographic Characteristics of Spotted Owls in the

Oregon Coast Ranges, 1990–2015 Technical Report. Corvallis, OR, USA: USDA Forest Service, Pacific Northwest Research Station.

Murray, J. (2007). *Mathematical Biology: I. An Introduction*. Interdisciplinary Applied Mathematics. Springer New York.

Wiens, J. D., Anthony, R. G., and Forsman, E. D. (2014). Competitive Interactions and Resource Partitioning Between Northern Spotted Owls and Barred Owls in Western Oregon. *Wildlife Monographs*, 185(1):1–50.

Table S1: The annual proportion of surveyed northern spotted owl (NSO) sites inhabited by NSO and barred owl (BAOW) within the Oregon Coast Range in the Pacific Northwest, USA. We obtained the proportions shown in the NSO and BAOW columns by dividing the NSO and BAOW sites by the total sites surveyed, respectively.

| Year | Sites Surveyed | NSO sites | BAOW sites | NSO | BAOW |
|---|---|---|---|---|---|
| 1990 | 141 | 110 | 3 | 0.780 | 0.021 |
| 1991 | 141 | 124 | 7 | 0.879 | 0.050 |
| 1992 | 165 | 128 | 10 | 0.776 | 0.061 |
| 1993 | 166 | 132 | 16 | 0.795 | 0.096 |
| 1994 | 170 | 141 | 14 | 0.829 | 0.082 |
| 1995 | 177 | 130 | 11 | 0.734 | 0.062 |
| 1996 | 186 | 136 | 20 | 0.731 | 0.108 |
| 1997 | 184 | 133 | 26 | 0.723 | 0.141 |
| 1998 | 194 | 145 | 39 | 0.747 | 0.201 |
| 1999 | 193 | 141 | 41 | 0.731 | 0.212 |
| 2000 | 200 | 134 | 55 | 0.670 | 0.275 |
| 2001 | 202 | 131 | 74 | 0.649 | 0.366 |
| 2002 | 204 | 130 | 77 | 0.637 | 0.377 |
| 2003 | 204 | 124 | 91 | 0.608 | 0.446 |
| 2004 | 204 | 114 | 92 | 0.559 | 0.451 |
| 2005 | 204 | 107 | 101 | 0.525 | 0.495 |
| 2006 | 204 | 105 | 124 | 0.515 | 0.608 |
| 2007 | 203 | 102 | 121 | 0.502 | 0.596 |
| 2008 | 203 | 82 | 134 | 0.404 | 0.660 |
| 2009 | 173 | 70 | 125 | 0.405 | 0.723 |
| 2010 | 172 | 71 | 115 | 0.413 | 0.669 |
| 2011 | 172 | 55 | 130 | 0.320 | 0.756 |
| 2012 | 172 | 57 | 140 | 0.331 | 0.814 |
| 2013 | 172 | 56 | 144 | 0.326 | 0.837 |
| 2014 | 172 | 48 | 124 | 0.279 | 0.721 |
| 2015 | 172 | 41 | 153 | 0.238 | 0.890 |

Note: These data have been sourced from the Appendix A of the 2016 annual USDA Forest Service report by Lesmeister et al. [2016].

Table S2: Model selection criteria used to compare one (Eq. 5) and two-way (Eq. 6) co-occurrence effects models to the classical Lotka-Volterra competition model (Eq. 4).

| Model | K | Log-Likelihood | BIC | $AIC_c$ | $\Delta\ AIC_c$ | $w_i(AIC_c)$ |
|---|---|---|---|---|---|---|
| One-way co-occurrence effect model (Eq. 5) | 5 | 102.799 | -182.145 | -191.644 | 0 | 0.801 |
| Two-way co-occurrence effects model (Eq. 6) | 6 | 102.747 | -178.109 | -188.827 | 2.817 | 0.196 |
| Classic Lotka–Volterra (Eq. 4) | 4 | 96.065 | -172.570 | -180.766 | 10.878 | 0.003 |

Note: $\Delta\ AIC_c$ represents the difference between corrected Akaike Information Criterion value $AIC_c$ of the specified model and the top performing model. The log-likelihood, number of parameters ($K$), Bayesian Information Criterion (BIC) and respective model weights ($w_i(AIC_c)$) are also provided.

Table S3: Model evaluation criteria and associated mathematical formulas used in evaluating modified Lotka-Volterra (L-V) models to estimate northern spotted owl (NSO) and barred owl (BAOW) population growth rates along the Oregon Coast, USA 1990 - 2015.

| Model evaluation criterion | Calculation |
|---|---|
| Likelihood ratio test | $LRT = -2\{\ln[L(\hat{\theta}_p \vert y)] - \ln[L(\hat{\theta}_{p+q} \vert y)]\} \sim \chi^2_q$ |
| Akaike information criterion (AIC) | $AIC = -2\ln[L(\hat{\theta}_p \vert y)] + 2p$ |
| Corrected Akaike Information Criterion $(AIC_c)$ | $AIC_c = -2\ln[L(\hat{\theta}_p \vert y)] + 2p\frac{n}{n-p-1}$ |
| Bayesian information criterion (BIC) | $BIC = -2\ln[L(\hat{\theta}_p \vert y)] + p.\ln(n)$ |

Note: The models evaluated accounted for potential negative effects of BAOW on NSO growth rates. Notation in Table S3 are defined as follows: $n$ is the number of data points; $p$ is a number of free within the model; $q$ is the additional parameter; $y$ is the observed data; $L$ represents the likelihood function (Johnson and Omland [2004]).

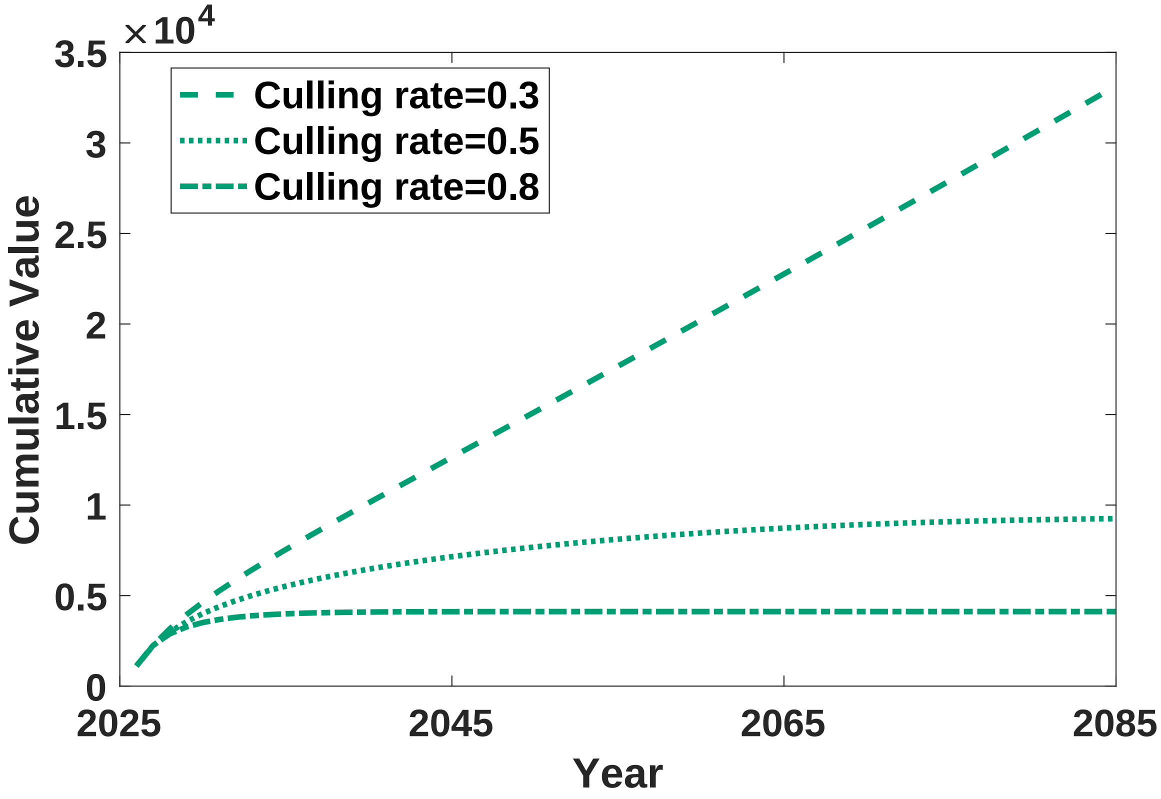


Figure S1: Cumulative plot showing the cumulative culling values for different culling rates. The values are calculated by multiplying the proportion of northern spotted owl (NSO) sites occupied by barred owls (BAOW) by 204 (the maximum number of sites surveyed within the study area), followed by cumulative summation. The final values are then multiplied by 6 individuals, given evidence that a single NSO site may support 2-4 BAOW territories (Wiens et al. [2014]) and assuming each BAOW territory would support a pair of individuals.
